# Écologie bactérienne de l'infection nosocomiale au service de réanimation de l'hôpital Laquintinie de Douala, Cameroun

**DOI:** 10.11604/pamj.2013.14.140.1818

**Published:** 2013-04-09

**Authors:** Clotilde Njall, Dieudonné Adiogo, André Bita, Noel Ateba, Gérald Sume, Basile Kollo, Fidèle Binam, Romain Tchoua

**Affiliations:** 1 Faculté de Médecine et des Sciences Pharmaceutiques de Douala, Université de Douala, BP 2701 Douala, Cameroun; 2 Faculté de Médecine et des Sciences Biomédicale, Département d'anesthésie réanimation, Université de Yaoundé 1, BP 13403 Yaoundé, Cameroun; 3 Hôpital d'Instruction des Armées Omar Bongo Ondimba –Libreville BP 20404, Gabon; 4 Délégation régionale de la santé Publique du Littoral, BP 106 Douala, Cameroun

**Keywords:** Bactéries, Douala, infection nosocomiale, réanimation, Bacteria, Douala, nosocomial infection, reanimation

## Abstract

**Introduction:**

L'objectif principal de notre étude était d'identifier les bactéries associées à l'infection nosocomiale, dans le service de réanimation, de l'hôpital Laquintinie de Douala en vue d'améliorer la prise en charge et diminuer la létalité.

**Méthodes:**

Il s'agissait d'une étude transversale et descriptive, menée du 1^er^ mars au 31 mai 2011.Tous les patients hospitalisés depuis au moins 48 h étaient inclus dans l’étude et ceux présentant une infection documentée à l'admission étaient exclus. L'analyse des donnés a été faite par le logiciel SPSS 16.Les tests de Khi deux pour la signification.

**Résultats:**

La prévalence de l'infection nosocomiale était de 12%, elle concernait des personnes âgées de plus de 60 ans et présentant une infection urinaire dans 79% des cas. La létalité était de 72% pour une durée moyenne de séjour de 11,7 ± 12,1 jours. Les bactéries responsables étaient en majorité des bactéries gram positifs (BGN), dont *E coli* dans 23,1% et les cocci gram positifs(CGP), dans 15,4% des cas.

**Conclusion:**

L’étude de la résistance aux antibiotiques, montre une multi résistance, dont il faut tenir compte en mettant en place une stratégie de prévention active.

## Introduction

Les infections nosocomiales sont des infections contractées dans un établissement de soins. [1] Cette définition a été revue en 2007 au vu de la diversification des structures et parcours de soins; et la survenue parfois tardive de l'infection après chirurgie [2]. L'infection nosocomiale peut être causée par les germes du patient, du personnel soignant ou de l'environnement hospitalier. Ces infections augment la morbidité, la mortalité, et le cout des soins à l'hôpital. [3] Aux Etats-Unis, on estime que les infections nosocomiales sont responsables de 9000 décès par an [4].

Bien que des efforts soient fournis pour renforcer les normes d'hygiènes dans les différents services hospitaliers, les infections nosocomiales continuent de persister dans les services de réanimation [5]. L'enquête nationale de prévalence de 2006 en France montrait une prévalence nationale des patients infectés de 4,97%. [6] En réanimation le nombre de patients infectés est plus élevé en raison de la densité des soins, de l'exposition à divers dispositifs invasifs et la gravité des pathologies des patients. Dans le réseau Réa Raisin 2007 en France la prévalence de l'infection nosocomiale était de 14,1% [7]. Au service de réanimation de l'hôpital IBN ROCHD de Casablanca le taux de prévalence de l'infection nosocomiale était de 17,9% [8].

Au Cameroun l'infection nosocomiale reste préoccupante en termes de morbidité et de mortalité, et plusieurs études y ont été consacrées comme celle de Simeu qui a analysé des mesures d'asepsie simples pour remédier à l'infection nosocomiale post -opératoire [9]. La présente étude effectuée au service de réanimation polyvalente de l'hôpital Laquintinie de Douala a pour objectif d'identifier les bactéries associées à l'infection nosocomiale; afin d'améliorer la prise en charge et réduire la morbidité et la létalité dans notre service.

## Méthodes

**Cadre et type d’étude:** Il s'agissait d'une étude descriptive transversale. Cette étude s'est déroulée dans le service de réanimation polyvalente de L'Hôpital Laquintinie de Douala (HLD) - Cameroun. Ce service a une capacité de 15lits; un ratio infirmiers/ lits > 0.5. Le plateau technique est insuffisant. L’ HLD d'une capacité de 745 lits reçoit la majorité des urgences de la ville d e Douala et de la sous région. L’étude a été menée sur une période de trois mois du 1^er^ mars au 31 mai 2011.

**Population d’étude:** La population concernait les patients admis au service de réanimation polyvalente pendant la période de l’étude, Tous les patients hospitalisés pendant au moins 48h étaient inclus dans l’étude. Les patients exclus de l’étude étaient ceux qui présentaient une infection documentée à l'admission

**Echantillonnage:** Elle était de convenance sur un mode non probabiliste. Tous ceux qui remplissaient les critères d'inclusions étaient recrutés.

**Matériel:** matériel de prélèvements et d'analyse bactériologiques des urines, sang, peau et bronches.

**Procédure de collecte des données:** Les informations étaient recueillies sur des fiches de questionnaires anonymes. Elles concernaient les données démographiques: (âge, sexe). Les données générales et cliniques concernaient les pathologies d'admission, les tableaux cliniques infectieux (le Syndrome Inflammatoire de Réponse Systémique (SIRS), le SEPSIS, le SEPSIS sévère). Le site de l'infection nosocomiale, le statut sérologique du Virus d'Immunodéficience Humaine (VIH), la provenance du patient (domicile, autres formations sanitaires.), la durée de séjour, le décès étaient aussi collectés. Les données biologiques étaient les résultats d'analyses de prélèvements bactériologiques (urine, peau, et sang). L'Examen Cytobactériologique des Urines (ECBU) était systématique chez les patients porteurs d'une sonde à demeure. Les prélèvements cutanés étaient réalisés sur des écoulements/suppurations cutanées. Les hémocultures étaient orientées en fonction du tableau clinique infectieux. Les prélèvements bronchiques ont été limités par le manque de matériel.

Le type de prélèvement et l'analyse bactériologique se faisaient en fonction du site de l'infection nosocomiale selon la classification du Comité de Coordination de Lutte contre l'Infection Nosocomiale (CCLIN) Paris-Nord en 1995 [10]. Seuls les examens positifs confirmaient l'infection nosocomiale dans notre série.

Des milieux sélectifs étaient utilisés en fonction des germes suspectés: Chapman pour les Staphylocoques, Gélose Eosine-Bleu de Méthylène (EMB) pour les bacilles Gram négatifs, Gélose chocolat pour les anaérobies et les Streptocoques, La gélose Muller Hinton pour les Antibiogrammes.

Le taux de résistance des bactéries isolés a été évalué par des tests successifs et systématiques sur 18 disques d'antibiotiques: les bectalactamines (Amoxicilline-Acide Clavulanique, Ceftazidim, Ceftriaxone, Cefuroxime, Oxacilline, Aztréonam, Imipénème, Pipéracilline, Tazobactam, Ticarcilline); lesAminosides (Amikacin, Neltimicine, Gentamicine, Tobramycine); les macrolides(Clindamycine); les quinolones (Ciprofloxacine, ofloxacine); les glycopeptides (Vancomycine); la Polymyxine(Colistine).

Les tableaux cliniques infectieux étaient évalués comme suit: Recherche du SIRS définit par deux des quatre critères suivants: température < 383°C ou 90/mn; fréquence respiratoire > 20/mn; globule Blancs >12000/mm^3^ ou < 4000/mm^3^.

Recherche d'un SEPSIS définit par l'association du SIRS et un site de l'infectiontieux (respiratoire, urinaire, cutané, digestif, méningée); recherche d'un SEPSIS sévère et ou choc septique définit par la présence d'un des signes de gravité suivant: acidose métabolique, trouble de la conscience, oligurie, trouble de la coagulation, défaillance cardiovasculaire.

**Ethique et confidentialité:** le protocole de l’étude a été soumis à la commission d’éthique de la Délégation Régionale de la Santé Publique du Littoral avec obtention de l'autorisation. La confidentialité des informations obtenues sur les sujets de l’étude a été respectée, car les noms des patients n'apparaissaient pas sur les documents de l’étude. Des sujets inclus dans l’étude ou leurs tuteurs et parents ont été renseigné pour obtenir leur consentement éclairé.

**Analyse statistique:** l'analyse des résultats était faite par le logiciel SPSS 16. Le test de khi deux pour les variables qualitatives et une valeur de p< 0,05 était considérée comme significative. L'erreur était fixée à 5%.

## Résultats

**Données - démographique de la population:** Nous avions 90 patients. Les hommes étaient 49(54,4%), avec un sexe -ratio Homme: Femme de 1,2. L’âge moyen était de 49,6 ± 1,8ans avec des extrêmes de 7 et 89 ans. Les patients âgés de plus de 60 ans étaient la tranche d’âge la plus concernée 32,9% (Tableau 1).

**Tableau 1:** Répartition des sujets selon l’âge et le sexe

Caractéristiques	Catégories	Effectifs	Pourcentage (%)
**Tranche d’âge**	0- 15ans	3	3,5
	16-30ans	14	16,5
	31- 45ans	15	17,5
	46-60ans	25	29,4
	≥60ans	28	32,9
**Sexe**	Homme	49	54,4
	Femme	41	45,6

**Prévalence de l'infection nosocomiale:** sur 90 patients, 11 ont présenté une infection nosocomiale soit une prévalence de patients infectés de 12%.

**Données cliniques:** sur les 11 patients infectés, 7(63,6%) présentaient les symptômes systémiques non spécifiques à type de SIRS, 3(27,6%) des SEPSIS. Sur les patients infectés, les sites d'infection recensés étaient urinaires 11(79%), cutanés 2 (17%), sanguin1 (7%) (Tableau 2). Le délai moyen d'apparition des signes cliniques infectieux était de 4,4 ± 3,2 jours avec des extrêmes de 2 et 14 jours. La létalité des patients infectés était de 8(72,7%).

**Tableau 2:** Répartition des sujets selon les types d'infection nosocomiale et tableau clinique des patients infectés

Caractéristiques	Catégories	Effectifs	Pourcentage (%)
**Type d'infection nosocomiale**	Infection urinaire	11	78,6
	Infection cutanée	2	14,3
	Bactériémie	1	7,1
**Tableau clinique**	SIRS	7	63,6
	SEPSIS	3	27,3
	Infection respiratoire	1	9,1

**Données générales:** la majorité des patients venaient du domicile 64(71,1%), suivis des patients référés d'autres formations sanitaires 11(12,2%), on ne notait pas une corrélation significative entre la provenance des patients et la survenue de l'infection nosocomiale (p = 0,766).

La durée moyenne d'hospitalisation était de 11,7 ± 12,1 jours chez les patients infectés avec des extrêmes de 3 et 45 jours. Les patients non infectés avaient une durée moyenne d'hospitalisation de 4,7 ± 3,1 jours avec des extrêmes de 2 et 25 jours.La durée moyenne d'hospitalisation était plus longue chez les patients infectés (pTableau 3).

**Tableau 3:** Répartition de l'infection selon certaines caractéristiques

Caractéristiques	Catégories	Infection				p
		Oui		Non		
		Effectifs	%	Effectifs	%	
Tranche d’âge	0-15ans	1	9,1	2	2,7	<0,00
	16-30ans	0	0	14	18,9	
	31- 45ans	2	18,2	13	17,6	
Statut HIV	46-60ans	3	27,3	22	29,7	0,379
	≥60ans	5	45,4	23	31,1	
	Négatif	9	81,8	45	71,4	
	Positif	2	18,2	18	28,6	
Durée moyenne d'hospitalisation	4,6 (2-25jours)	0	0	79	87,8	<0,004
	11,7 (3-45jours)	11	12,2	0	0	
Pathologieà l'admission	Pathologie médicale	55	68,8	8	72,7	0,901
	Pathologie chirurgicale	5	6,2	1	9,1	
	Traumatologie	20	25	2	18,2	
Provenance du patient	Domicile	8	73	56	70,9	0,766
	Autres formations sanitaires	2	18	9	11,4	
	Chirurgie	0	0	4	5	
	Autres services hospitalisation	1	9	10	12,7	

**Données biologiques:** sur les 11 patients infectés, 13 prélèvements biologiques ont été effectués isolant 11Bactéries Gram Négatifs(BGN), et 2 Cocci Gram Positifs(CGP) Les principales espèces bactériennes étaient Escherichia Coli 3(23,1%), suivi de Acinetobacter baumani 2(15, 4%), Pseudomonas aeruginosa 2(15,4%), et Staphylococcus aureus 2(15, 4%) (Tableau 4).

**Tableau 4:** Bactéries associées à l'infection nosocomiale

Bactéries	Fréquence	Pourcentage (%)
*Acinetobacter baumani*	2	15,4
*Citrobacter Koseri*	1	7,6
*Echerichia coli*	3	23,1
*Klebsiella oxycota*	1	7,7
*Providencia stuarti*	1	7,7
*Pseudomonas aeruginosa*	2	15,4
*Pseudomonas florescens*	1	7,7
*Staphylococcus aureus*	2	15,4
**Total**	**13**	**100**

Sur 18 disques d'antibiotiques testés, *Pseudomonas aeruginosa* avait le taux de multi résistance le plus élevé (88,8%). *Pseudomonas fluorescens* avait le taux le plus bas (44,4%). *Acinetobacter baumani* (61,1%), *Staphylocoques aureus* (61,1%) et *Escherichia coli* (66,7%) avaient des taux de multi résistance aussi élevés (Tableau 5).

**Tableau 5:** Taux de résistance des bactéries sur 18 disques d'antibiotiques testés

		Resistant		Sensible		Total	
No	Bactéries	N	%	N	%	N	%
**1**	*Pseudomonas florecens*	8	44,4	10	55,6	18	100,00
**2**	*Acinetobacter baumanii*	22	61,1	14	38,9	36	100,00
**3**	*Staphylococcus aureus*	22	61,1	14	38,9	36	100,00
**4**	*Citrobacter koseri*	12	66,7	6	33,3	18	100,00
**5**	*Escherichia coli*	36	66,7	18	33,3	54	100,00
**6**	*Klebsiella oxycota*	13	72,2	5	27,8	18	100,00
**7**	*Provindencia stuartti*	14	77,8	4	22,2	18	100,00
**8**	*Pseudomonas aeruginosa*	32	88,9	4	11,1	36	100,00

N = Nombre,% = Pourcentage

**Profil de sensibilité:** *Pseudomonas aeruginosa* présentait 100% de résistance avec la Ceftazidime, la Tircacilline, la Ceftriaxone, les aminosides, et quinolones testés. Cependant l'Imipénème reste très actif avec 100% de sensibilité. *Acinetobacter baumani* avait 50% de résistance avec la Ceftazidime, la ticarcilline, l'Imipénème, les aminosides, et les autres B lactamines testés. Les quinolones restent très actives avec 100% de sensibilité. *Staphylocoque aureus* montrait 50% de résistance avec l'Oxacilline, la Vancomycine, et la Gentamycine. L'Amikacin et la Neltimicine restent très actifs avec 100% de sensibilité. *Escherichia coli* avait 100% de résistance avec la Céftriaxone, la Vancomycine et les quinolones testés. l'Amoxicilline - Acide clavulanique présentait 66,66% de résistance. Une résistance de 33,33% a été notée avec la ceftazidime. L'association Pipéracilline-Tazobactam, L'Imipénème, et l'Amikacine restent très actifs avec 100% de sensibilité (Tableau 6).

**Tableau 6:** Profil de sensibilité des bactéries

	Germes							
Antibiotiques	Acinetobacter baumanii	Pseudomonas aeruginosa	Provindencia stuartti	Klebsiella oxycota	Staphylococcus aureus	Pseudomonas florecens	Citrobacter koseri	Escherichia coli
Amoxicilline + Acide Clavulanique	0%	0%	0%	0%	50%	0%	0%	33,33%
Ticarcilline	50%	0%	0%	0%	50%	100%	0%	0%
Piperacilline + Tazobactam	50%	0%	100%	0%	50%	100%	100%	100%
Imipeneme	50%	100%	0%	100%	50%	100%	100%	100%
Cefuroxime	50%	0%	0%	0%	50%	0%	0%	0%
Ceftriaxone	0%	0%	0%	0%	0%	0%	0%	0%
Ceftazidime	50%	0%	0%	0%	0%	100%	0%	66,66
Amikacine	50%	0%	100%	100%	100%	100%	0%	100%
Gentamicine	50%	0%	0%	0%	50%	0%	0%	0%
Netilmicine	50%	0%	0%	0%	100%	0%	0%	66,66%
Tobramicine	50%	0%	0%	0%	50%	100%	0%	33,33%
Ciprofloxacine	100%	0%	100%	100%	0%	100%	100%	0%
Ofloxacine	100%	0%	0%	100%	0%	100%	100%	0%
Astreonam	0%	0%	100%	0%	0%	100%	100%	33,33%
Clindamycine	0%	0%	0%	0%	50%	0%	0%	0%
Colistine	50%	100%	0%	100%	0%	100%	100%	66,66%
Vancomycine	0%	0%	0%	0%	50%	0%	0%	0%
Oxacilline	0%	0%	0%	0%	50%	0%	0%	0%
**Effectif**	**2**	**2**	**1**	**1**	**2**	**1**	**1**	**3**

## Discussion

Au terme de notre étude sur l'infection nosocomiale dont l'objectif était d'identifier les bactéries associées à l'infection nosocomiale en réanimation; en vue d'améliorer la prise en charge; il ressort que les bactéries fréquemment rencontrées étaient des BGN. Ces bactéries étaient multi résistantes aux différentes familles d'antibiotiques testés.

La prévalence de l'infection nosocomiale de 12% dans notre série se rapproche de ceux des services de réanimation du Réseau Raisin 2007 en France qui était de 14,4% [5, 7]. Des taux plus élevés ont été retrouvés dans d'autres études en réanimation, comme (22,1%) dans le réseau REA SUD EST [11]. Ceci pouvait s'expliquer par les différences des caractéristiques de la population de l’étude, qui était jeune dans notre série avec un âge moyen de 49,6 ± 1,8ans et les pathologies de gravité différentes. La pathologie médicale était plus retrouvée dans notre série. Le plateau technique insuffisant limitait les gestes invasifs de suppléance cardio-pulmonaire. Brun-Buisson, recommande la substitution des procédures invasives par des procédures moins à risque chaque fois que cela est possible. [12] Les techniques de collecte et d'analyse bactériologique utilisées dans notre série étaient différentes des autres études. Dans un essai randomisé, cette différence de technique peut varier le taux d'infection du simple au double [13].

Les patients de plus de 60 ans étaient plus exposés à l'infection nosocomiale dans notre série (32,9%), avec une augmentation du risque de survenue de l'infection nosocomiale. Ceci pourrait s'expliquer par la diminution des défenses immunitaires et l'existence des co morbidités présents à cet âge. Ce résultat est comparable à celui d'Haley et al, qui montre que le risque d'infection nosocomiale augmente avec l’âge [14].

Sur le plan clinique les patients infectés présentaient des symptômes systémiques aspécifiques avec 63,6% de SIRS. La fréquence des formes asymptomatiques en particulier chez les patients sondés peuvent être méconnues si un ECBU systématique n'est pas pratiqué. Escherichia coli est le micro-organisme le plus souvent isolé dans 20% des cas. [15] Dans notre série tous les patients infectés étaient sondés. Escherichia coli a été isolé dans 23,1% des cas. La proportion des sites d'infection nosocomiale varie suivant l'activité du service [2, 10]. Dans notre série le site urinaire était fréquent lié à l'exposition élevée des patients au sondage urétral. Une faible exposition des patients à la ventilation mécanique et au cathétérisme veineux et artériel, expliquerait le faible taux de pneumopathie et bactériémie/septicémie de notre série. Les patients opérés ne séjournait pas plus de trois jours dans notre service ce qui n'a pas permis d'observer les infections du site opératoire dans notre série. Pour Richards et al au Etats ‘Unis, les infections urinaires représentaient (31%) des infections nosocomiales suivies par les pneumopathies (27%) et les bactériémies (19%). [16]

La durée d'hospitalisation était plus longue chez les patients infectés. Ce résultat est comparable à une étude multicentrique canadienne, qui montrait que la durée du séjour augmentait en moyenne de 4,3 jours chez les patients infectés [13].

Le délai moyen d'apparition des signes infectieux était de 4,4 ± 3,2 jours. Ainsi un séjour supérieur à 4jours dans notre service serait un facteur de risque de survenu d'une infection nosocomiale. La létalité de 72,7% des patients infectés reste très élevée. Le risque d’échec thérapeutique est très élevé du fait de la multi résistance de bactéries rencontrées, aux antibiotiques utilisés. Par ailleurs les antibiotiques sensibles sont onéreux rendant l'accès difficile au traitement. Ballow et al. parlent d'impasse thérapeutique [17]. Ceci montre la nécessité de mettre dans nos services les mesures de prévention dont l'efficacité est reconnue; en insistant sur l'organisation et le renforcement des moyens matériels et humains appropriés [18]. Un ratio infirmières: patients > 0,5 accroit de façon sensible le risque d'infection croisée [19].

Il est difficile d'imputer la létalité à la seule infection nosocomiale. Certains facteurs comme la gravité des pathologies d'admission doivent être pris en compte. [20] En effet une étude française, ne montrait pas de surmortalité significative de ces infections après ajustement sur la gravité des malades avant la survenue de l'infection [21].

Les bactéries liées à l'infection nosocomiales dans notre série étaient en majorité *Echerichia Coli, Pseudomonas aeruginosa, Acinetobacter baumanii, Staphylococcus aureus*. Ces bactéries sont fréquemment à l'origine de l'infection nosocomiale en réanimation [22]. Les proportions rapportées par Djerfaouir et al en Tunisie, étaient différents, *Pseudomonas aeruginosa* (34%), *Acinetobacter baumani* (20%) [23]. Ces bactéries peuvent être ceux des malades qui colonisent de nouveaux sites à la faveur d'un acte invasif ou une fragilité particulière. La provenance peut être exogène à la faveur des transmissions croisées (malade, personnel, environnement) [22]. Lucet et coll ont montré que 7% des malades admis en réanimation étaient porteurs du *Staphylococcus aureus* résistant à la mélticilline (SARM) [24].

Les bactéries rencontrées étaient multi résistantes parce que du fait de l'accumulation des résistances naturelles et ou acquises, elles n’étaient sensibles qu’à un petit nombre d'antibiotiques habituellement actifs en thérapeutique. (25) Siah au Maroc a rapporté un taux de multi résistance de 60% avec *Pseudomonas aeruginosa* et *Acinetibacter baumani* [26]. Les raisons reposent principalement sur la pression de sélection exercée par les antibiotiques et la diffusion des souches résistantes [5, 27].

Dans notre série, le profil de sensibilité des bactéries montrait des proportions élevée de bactéries Mutirésistants aux antibiotiues (BMR) comme des pseudomonas résistants à la Ceftazidime(PARC), des *Staphylocoques aureus* résistants à la méticillin(SARM), et aux glycopeptides(GISA). Des entérobactéries résistantes aux céphalosporines de 3^ème^ génération(ERC), et de *Acinétobacter baumani* multirésistant.

Une étude réalisée en 1990-91 dans 10 pays européens et portant sur la proportion de SARM parmi les souches de *S. aureus*, a permis d'opposer deux groupes de pays: l'Italie, la France, l'Espagne, où la proportion de SARM est très élevée ( 30%), et le Danemark, la Suède, les Pays-Bas où la proportion de SARM est très basse ( 2%). A la même époque, une étude sur la résistance des bacilles à Gram négatif isolés dans les unités de soins intensifs a montré que la proportion de souches de *Klebsiella pneumoniae* résistantes aux céphalosporines de 3^ème^ génération par production de B lactamines à spectre élargie( BLSE) était beaucoup plus élevée en France (36%) qu'en Belgique (13%), Pays-Bas (12%) et Allemagne (5%), ainsi que celle des souches de Pseudomonas aeruginosa résistants à l'imipénème: 22% en France et 10 à 17% dans les 3 autres pays [22]. Des résultats d'enquête dans les hôpitaux français ont montré que des proportions élevées de souches multi résistantes connues pour être des marqueurs de l'hygiène, comme celles du SARM, et des ERC, étaient le reflet de l'insuffisance ou du dysfonctionnement de l'organisation de la lutte contre les infection nosocomiale dans les hôpitaux [28]. Dans notre série la prescription anarchique des antibiotiques et la transmission croisée des germes liés aux non respect des règles d'hygiène pourraient expliquer cette multi résistance. Il serait souhaitable de mettre dans notre service une politique active de lutte contre les BMR. Celle-ci repose, en premier lieu, sur l'application et le strict respect, pour tout patient, des précautions d'hygiène “standard” lors de soins potentiellement contaminants [29]. Des mesures d'isolement complémentaires, adaptées aux modes de transmission du germe en cause, sont à appliquer lorsqu'une BMR est suspectée ou identifiée chez un patient. En particulier, il faut souligner l'importance de la signalisation des patients porteurs de BMR et de la transmission de l'information aux services ou établissements qui reçoivent ces patients lors d'un transfert et d'une hospitalisation. Selon les recommandations du comité de coordination de lutte contre l'infection nosocomiale paris nord [30].

## Conclusion

L'infection nosocomiale était plus élevée chez les patients âgés. La durée d'hospitalisation était plus longue chez les patients infectés. La prévalence de l'infection nosocomiale était de 12% et la létalité des patients infectés de 72%. Les bactéries fréquemment liées à l'infection dans notre série étaient, des BGN dont Escherichia coli, et des CCP dont *Staphylococcus aureus*. Ces bactéries étaient multi résistantes. Il serait souhaitable de mettre dans notre hôpital une politique globale de prévention des infections nosocomiales qui repose, en particulier, sur le respect par le personnel de précautions d'hygiène; complétée d'une stratégie active de maîtrise de la dissémination des BMR qui, pour être efficace, doit s'inscrire dans la durée et la continuité, et s'appuyer sur la mobilisation de l'ensemble des professionnels de santé. Une étude ultérieure sur le portage des BMR dans notre service nous permettra de préciser l'origine des bactéries et leur profil de résistance.
